# Pathophysiology, Management and Quality of Life in Atopic Dermatitis and Psoriasis—A Challenge for Patients and Their Families

**DOI:** 10.3390/children9050592

**Published:** 2022-04-22

**Authors:** Michele Callea

**Affiliations:** Pediatric Dentistry and Special Dental Care Unit, Meyer Children’s Hospital, 50139 Florence, Italy; michele.callea@meyer.it or mcallea@gmail.com

In this Special Issue of *Children*, we present two articles: one detailing the pathophysiology of atopic dermatitis (AD) and psoriasis and highlighting recommendationsin regard to the implications for management in children [1], and the other reviewing and analyzing the quality of life of these patients and their families [2]. In studies of AD and psoriasis, chronic inflammatory skin conditions have been associated with a significant cutaneous and systemic disease as well as a poor health-related quality of life. Recently, the complex pathophysiologies of both AD and psoriasis have been reviewed: this article discusses the implications for treatment with current state-of-the-art and emerging topical and systemic therapies [1]. Both AD and psoriasis are caused by a complex combination of immune dysregulation, skin-barrier disruption, genetic factors, and environmental influences [1]. Previous treatments for both diseases were limited to anti-inflammatory agents that broadly suppress inflammation. Novel relevant pathways, including recognition of the role of T-helper type 2-driven inflammation in AD and T-helper 1- and 17-driven inflammation in psoriasis, have led to a therapeutic revolution [1]. As stated in the original research article, there are a number of novel treatment options available for AD and psoriasis, with many more currently under investigation. Furthermore, the review article examines the quality of life (QoL) of children with AD and psoriasis since they both negatively impact patients in multiple aspects such as physical, psychosocial, and mental functioning [2]. In these two articles, attention is given to AD in relation to other disorders and to psychosocial/mental comorbidities; specific sections describe the impact of comorbidities on QoL. To create strategies to improve QoL, a multidisciplinary approach, including communication and collaboration between primary care providers, pediatric specialists, dermatologists, psychiatrists, nurses, social workers, nutritionists, and support groups, is essential for long-term control of these disorders. When psychological support is needed, physicians should refer their patients to a mental health specialist when necessary and explain possible comorbidities to the patient as part of the management plan [1].

Validated instruments designed to assess the QoL of patients provide an additional level of understanding to a patient’s condition and allow for a standardized longitudinal assessment of the therapeutic effects. A multidisciplinary approach may lead to a better understanding of the disease and therefore may improve a patient’s QoL. Additional development and evaluation of more practical QoL scales for exploring treatment efficacy as well as additional support strategies for pediatric patients and their families are needed, and these topics are widely discussed in these two articles [1,2].

An improved understanding of the pathophysiology of AD and psoriasis and comprehension on how to improve QoL has led to a revolution in targeted therapy. As additional novel agents are approved, the next goal in the management of AD and psoriasis will be the tailoring of specific treatments for individual patients. The next decade of research will be dominated by extensive genetic, molecular, and clinical phenotyping of patients in order to understand which pathologic mechanisms are most relevant [1,2].

Currently, stress-related disorders and skin or genetic diseases need more recommendations; thus, we believe that a review on the quality of life of patients affected by AD and psoriasis and a research article evaluating the pathophysiology of these diseases and focusing on their treatments would be of benefit for future personalized treatments [1,2].
